# Norcantharidin down-regulates iron contents in the liver and spleen of lipopolysaccharide-treated mice

**DOI:** 10.1080/13510002.2022.2088011

**Published:** 2022-06-23

**Authors:** Jie Zheng, Jiao-Jiao Wang, Hui-Min Ma, Meng-Qi Shen, Zhong-Ming Qian, Yu-Xin Bao

**Affiliations:** a Research Center for Medicine and Biology, Zunyi Medical University, Zunyi, People’s Republic of China; b Institute of Translational and Precision Medicine, Nantong University, Nantong, People’s Republic of China; c National Clinical Research Center for Aging and Medicine, Huashan Hospital, Fudan University, Shanghai, People’s Republic of China; d Laboratory of Neuropharmacology, Fudan University School of Pharmacy, Shanghai, People’s Republic of China

**Keywords:** Norcantharidin (NCTD), tissue iron, TfR1 and Fpn1, IRP1 and hepcidin, IL-6/JAK2/STAT3 signaling pathway, mice, liver, spleen

## Abstract

**Objective:**

The inhibiting effect of Norcantharidin (NCTD) on IL-6 (interleukin-6) and STAT3 and the involvement of the IL-6/STAT3 pathway in hepcidin expression prompted us to speculate that NCTD could affect iron metabolism.

**Methods:**

We examined the effects of NCTD on serum iron (SI) and transferrin (Tf) saturation, iron and ferritin light chain (FTL), transferrin receptor 1 (TfR1), divalent metal transporter 1 (DMT1), ferroportin 1 (Fpn1), iron regulatory protein 1 (IRP1) and hepcidin, as well as IL-6 and STAT3 in the liver, spleen and duodenum of mice treated with lipopolysaccharide (LPS) in vivo, using RT-PCR, Western blotting and immunofluorescence analysis.

**Results:**

NCTD could increase SI and Tf saturation and reduce tissue iron and FTL content by affecting expression of cell-iron transport proteins TfR1, DMT1 and Fpn1. The impact of NCTD on TfR1, DMT1 and Fpn1 expression is mediated by up-regulating IRP1 and down-regulating hepcidin expression, while NCTD-induced down-regulation of hepcidin is mediated by the IL-6/STAT3 signalling pathway in LPS-treated mice.

**Conclusions:**

NCTD affects iron metabolism by modifying the expression of IL-6/JAK2/STAT3/hepcidin and IRP1 and suggest that the ability of NCTD to reduce tissue iron contents may be a novel mechanism associated with the anti-cancer effects of NCTD.

## Introduction

Cantharidin (CTD) is a naturally occurring compound isolated from 1500 species of medicinal insect blister beetle (*Mylabris phalerata* Pallas) [1]. The use of mylabris as a traditional medicine in China can be traced back more than 2000 years, and it is still used as a folk medicine today [2]. The most important of the medicinal uses of CTD is its anti-cancer activities [2,3]. However, the application of CTD is limited due to its toxicity to the gastrointestinal and urinary tracts. Norcantharidin (NCTD) is a demethylated analog of cantharidin (CTD) [2]. NCTD causes fewer nephrotoxic and inflammatory side effects than CTD [4], and like CTD has been demonstrated as a potential agent against certain cancers including human prostate cancer, stromal cancer, non-small lung cancer and hepatocellular carcinoma through inhibiting proliferation and metastasis of tumor cells [2,5–8].

The inhibiting effect of NCTD on IL-6/JAK2/STAT3 (interleukin-6/Janus kinases 2/signal transducer and activator of transcription 3) signaling pathway [9–12] and the involvement of the IL-6/JAK2/STAT3 pathway in hepcidin regulation [13–15] prompted us to speculate that NCTD may modify iron metabolism by affecting the expression of hepcidin because hepcidin is a central player in maintaining body iron homeostasis [16–18].

In the present in vivo study, we examined the effects of NCTD on serum iron (SI) and transferrin (Tf) saturation, iron contents and ferritin-light chain (FTL) expression in the liver, spleen and duodenum of LPS-treated mice. To find out the reasons for the changes in serum and tissue iron contents induced by NCTD, we then investigated the effects of NCTD on the expression of the major cell-iron importers transferrin receptor 1 (TfR1) and divalent metal transporter 1 (DMT1), and the cell-iron exporter ferroportin 1 (Fpn1). To clarify the mechanisms underlying the impact of NCTD on cell-iron-transporters under inflammatory conditions, we dissected the changes in the expression of IRP1 and hepcidin as well as IL-6, p-JAK2 and p-STAT3 in mice co-treated with NCTD and LPS. We demonstrated that NCTD could increase SI and Tf saturation and reduce iron contents in the liver, spleen and intestine in LPS-treated mice by affecting both IRP and hepcidin expression, providing evidence for the effect of NCTD on iron metabolism.

## Materials and methods

### Materials

Unless otherwise stated, all chemicals were obtained from the Sigma Chemical Company, St. Louis, MO, USA. Mouse monoclonal anti-mouse TfR1 (cat.13-6800) was purchased from Invitrogen, Carlsbad, CA, USA; rabbit polyclonal anti-IRP1 (cat. ab236773), rabbit polyclonal anti-Fpn1 (cat. ab78066) and anti-DMT1 (cat. ab123085) from Abcam, Inc., Cambridge, UK; rabbit polyclonal anti-FTL (cat. 10727-1-AP) from Protein-tech, Chicago, IL, USA; mouse anti-p-JAK2 (cat. 3771), rabbit anti-JAK2 (cat. 3230), mouse anti-p-STAT3 (cat. 9145) and rabbit anti-STAT3 (Tyr705) (cat. 9139) from Cell Signaling Technology, Boston, USA; goat anti-rabbit or anti-mouse IRDye 800 CW secondary antibody (cat. 926-32210) from LI-COR Bio Sciences, Lincoln, NE, USA; FastStart Universal SYBR Green Master (cat. 11123) and AevertAid First Strand cDNA Synthesis Kit (cat. 11119ES60) from Yeasen BioTechnologies Co., Shanghai, China; LightCycler96 (cat. 05815916001) from Roche, Nutley, NJ, USA; TRIzol reagent (cat.15596018) from Life Technologies, Carlsbad, CA, USA; BCA protein Assay kit (cat. 23225) from Thermo Scientific, Waltham, MA, USA; and protein RIPA lysis buffer (cat. P0013B) from Beyotime Institute of Biotechnology, Haimen, JS, China.

### Animals and treatments

Forty-five balb/c male mice (18–22 g) were used in the present study. The mice were housed under specific pathogen-free conditions at 22 ± 2°C with a relative humidity of 60-65% and maintained under a 12-h light/12-h dark cycle with ad libitum access to food and water as previously described. All animal care and experimental protocols were performed according to the Animal Management Rules of the Ministry of Health of China and approved by the Animal Ethics Committees of Nantong University (NSFC31271132). The mice were randomly divided into three groups: (1) Control group: intraperitoneal injection (i.p.j.) of 0.2 ml normal saline at 8am for 3 days and then 0.4 ml normal saline on the third day; (2) LPS group: intraperitoneal injection of 0.2 ml normal saline at 8am for 3 days and then LPS (1mg/kg) in 0.4 ml normal saline at the third day; (3) NCTD + LPS group: intraperitoneal injection of NCTD (2 mg/kg) in 0.2 ml normal saline at 8am for 3 days and then LPS (1 mg/kg) in 0.4 ml normal saline at the third day. At 2pm on the third day (6-h after LPS treatment), animals were anesthetized with 1% (w/w) pentobarbital sodium (40 mg/kg body weight, intraperitoneally) and decapitated. The dose of the NCTD drug used was according to Liu et al. [19].

### Sampling of blood and tissues

After anesthetization and decapitation, blood samples were collected into heparinized syringes for the determination of SI, UIBC and TF% saturation. Mice were then perfused with phosphate-buffered saline (PBS), the liver, spleen and duodenum were removed, excised, and rinsed in PBS, weighed and stored in a freezing chamber at −80°C for total RNA extraction, protein determination, and iron measurement [20,21].

### Serum iron and transferrin saturation

SI and UIBC were measured using commercial kits (Pointe Scientific, cat. I7504-6) as described [22]. TIBC (micrograms per deciliter TIBC = SI + UIBC) and transferrin saturation (TS = SI/TIBC × 100) were calculated.

### Western blot analysis

The tissues were washed and homogenized by protein RIPA lysis buffer as described previously [23,24]. Soluble proteins were collected after centrifugation at 13200 rpm for 15 min at 4°C and protein content was determined using the BCA protein assay reagent kit. Aliquots of the extract containing about 40 μg of protein were loaded and run on a single track of 12% (v/v) (for FTL) and 10% (v/v) (for others) sodium dodecyl sulfate-polyacrylamide gel electrophoresis under reducing conditions before being subsequently transferred to a PVDF membrane. The blots were blocked with 5% (w/v) non-fat milk and then incubated overnight at 4°C with primary antibodies: mouse anti-TfR1 (1:500 dilution), rabbit anti-DMT1 (1:1000 dilution), rabbit anti-Fpn1 (1:1000 dilution), rabbit anti-Ft-L (1:1000 dilution), rabbit anti-IRP1 (1:1000 dilution), mouse anti-p-JAK2 (1:1000 dilution), rabbit anti-JAK2 (1:1000 dilution), mouse anti-p-STAT3 (1:1000 dilution), and rabbit anti-STAT3 (1:1000 dilution). After being washed three times, the blots were incubated with goat anti-rabbit (1:5000 dilution) or anti-mouse IRDye800 CW secondary antibody (1:5000 dilution) for 2-h at room temperature. The intensities of the specific bands were detected and analyzed by the Odyssey infrared imaging system (Li-Cor, Lincoln, NE, USA). Anti-β-actin (1:1000 dilution) was used as internal protein controls.

### Isolation of total RNA and quantitative real-time PCR

The extraction of total RNA and preparation of cDNA were performed using TRIzol reagent and the AevertAid First Strand cDNA Synthesis Kit respectively, in accordance with the instructions of the manufacturers. Real-time PCR was carried out by RT-PCR instrument (LC96, Roche, Switzerland) using Fast Start Universal SYBR Green Master and the Light Cycler96. The specific pairs of primers were: mouse β-actin, forward: 5′-AAA TCG TGC GTG ACA TCA AAGA-3′, reverse: 5′-GCC ATC TCC TGC TCG AAG TC-3′; mouse hepcidin, forward: 5′-AGA GCT GCA GCC TTT GCA C-3′, reverse: 5′-GAA GAT GCA GAT GGG GAA GT-3′; and IL-6, forward: 5′-CTG CAA GAG ACT TCC ATC CAG-3′, reverse: 5′-AGT GGT ATA GAC AGG TCT GTTGG-3′ (Supplementary Table 1). The CT values of each target gene were normalized to that of the β-actin mRNA. Relative gene expression was calculated by the 2^−ΔΔ^CT method [25,26].

### Immunofluorescence staining

Immunofluorescence staining was carried out as described previously [27,28]. Slices were blocked with 3% bovine serum albumin for 2 h and incubated overnight at 4°C with primary antibodies: mouse anti-TfR1 (1:250), rabbit anti-DMT1 (1:100), rabbit anti-Fpn1 (1:100), rabbit anti-FTL (1:100) and rabbit anti-IRP1 (1:200). After being washed with 0.01 M PBS three times, the slides were incubated with Alex Fluor 488 or 594-conjugated secondary antibodies (Thermofisher, cat.1853312; cat. 2043369) for 1 h at room temperature and then with DAPI (Vectashield, cat. H-1200) for 15 min at 37°C. Negative controls received an identical treatment except for the primary antibody and showed no positive signal.

### Tissue iron measurement

The total iron in the tissues (μg/g wet weight of tissue) was determined using a graphite furnace atomic absorption spectrophotometer as described [29]. In brief, tissues were homogenized in 20 mM 4-(2-hydroxyethyl)-1-piperazine ethanesulfonic acid, followed by digestion in an equal volume of ultrapure nitric acid, and then measured with a graphite furnace atomic absorption spectrophotometer (Perkin-Elmer; Analyst 100).

### Statistical analysis

Statistical analyses were performed using the Graphpad Prism 8.0. Data are presented as mean ± standard error of the mean. Differences between the means were analyzed using D’Agostino-Pearson omnibus normality test. For data not following normal distribution, Kruskal–Wallis test with Dunn’s multiple comparisons test was performed. A *p* < .05 denoted statistical significance.

## Results

### Norcantharidin increased serum iron, serum transferrin saturation, and reduced iron content in the liver, spleen and duodenum in lipopolysaccharides-treated mice

**Figure 1.**
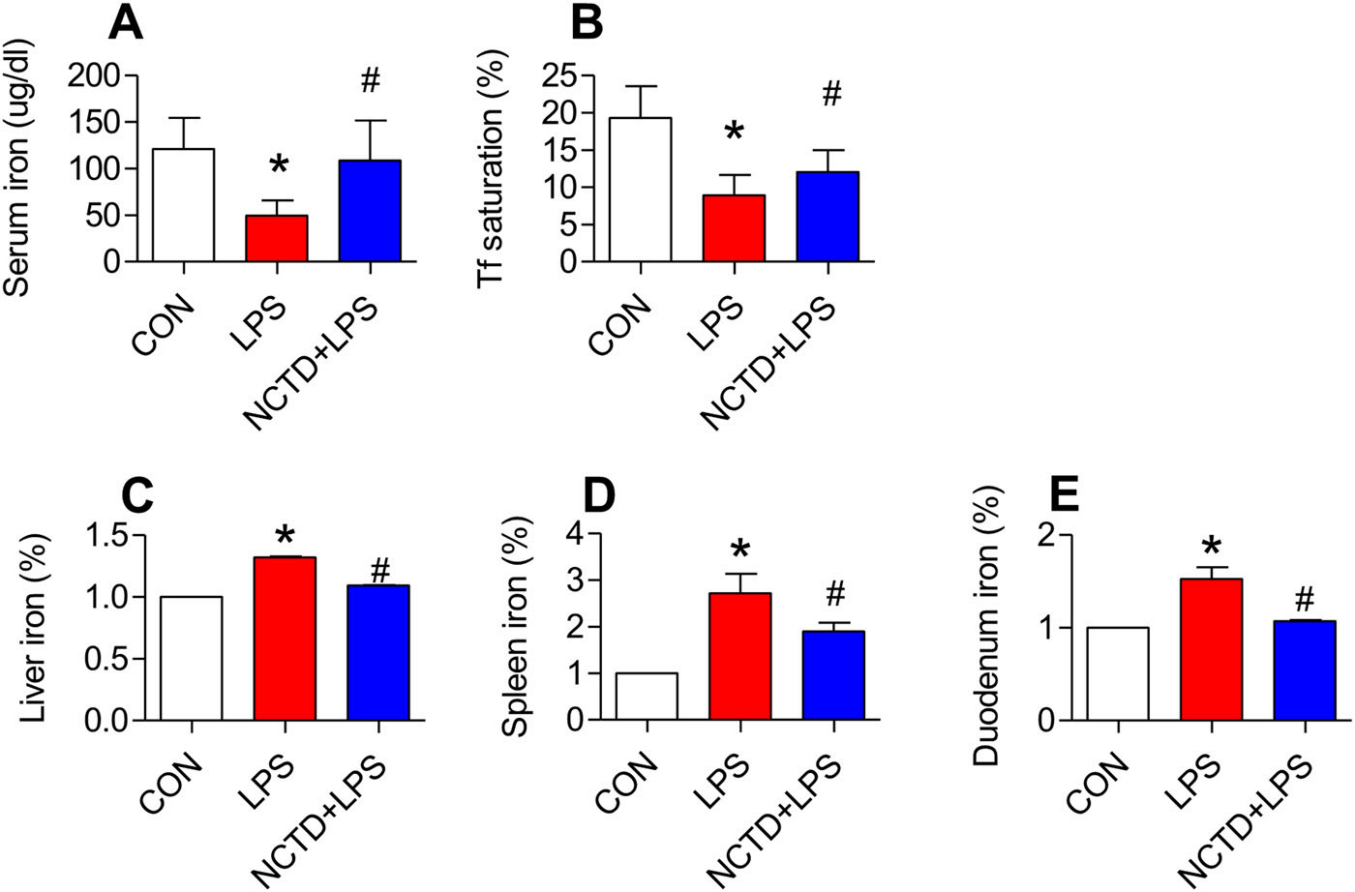
Norcantharidin increased serum iron, serum transferrin saturation, and reduced iron content in the liver, spleen and duodenum in lipopolysaccharides-treated mice. The mice in Control, LPS and NCTD + LPS groups were treated as described in ‘Methods’. At 6-h after treatment, the animals were anesthetized and decapitated, the samples collected and serum iron (A), transferrin saturation (B), UIBC (C), and iron contents (% Control) in the liver (D), spleen (E) and duodenum (F) were measured. Data presented as the means ± S.E.M. (n = 3) *p < .05 vs. Control. #*p* < .05 vs. LPS.

We first examined the effects of NCTD on serum iron (SI), transferrin saturation and UIBC, and iron contents in the liver, spleen and duodenum of LPS-treated mice. Treatment with LPS only induced a significant reduction in serum iron (Figure 1(A)) and transferrin saturation (Figure 1(B)), and an increase in iron content in the liver (Figure 1(D)), spleen (Figure 1(E)) and duodenum (Figure 1(F)) of mice, while pre-treatment with NCTD induced a significant reversion in these changes induced by LPS (Figure 1(A,B,D–F)). Serum iron (Figure 1(A)) and transferrin saturation (Figure 1(B)) were significantly higher, and iron content in the liver (Figure 1(D)), spleen (Figure 1(E)) and duodenum (Figure 1(F)) was lower in mice treated with NCTD + LPS when compared to mice treated with LPS only. There were no significant differences in UIBC among the control, LPS and NCTD/LPS groups (Figure 1(C)). The findings clearly showed that NCTD could up-regulate serum iron and transferrin saturation, but not UIBC, and down-regulated iron content in the liver, spleen and duodenum in LPS-treated mice.

### Norcantharidin up-regulated transferrin receptor 1, divalent metal transporter 1, and ferroportin 1 expression, and down-regulated ferritin-light chain expression in liver of lipopolysaccharides-treated mice

**Figure 2.**
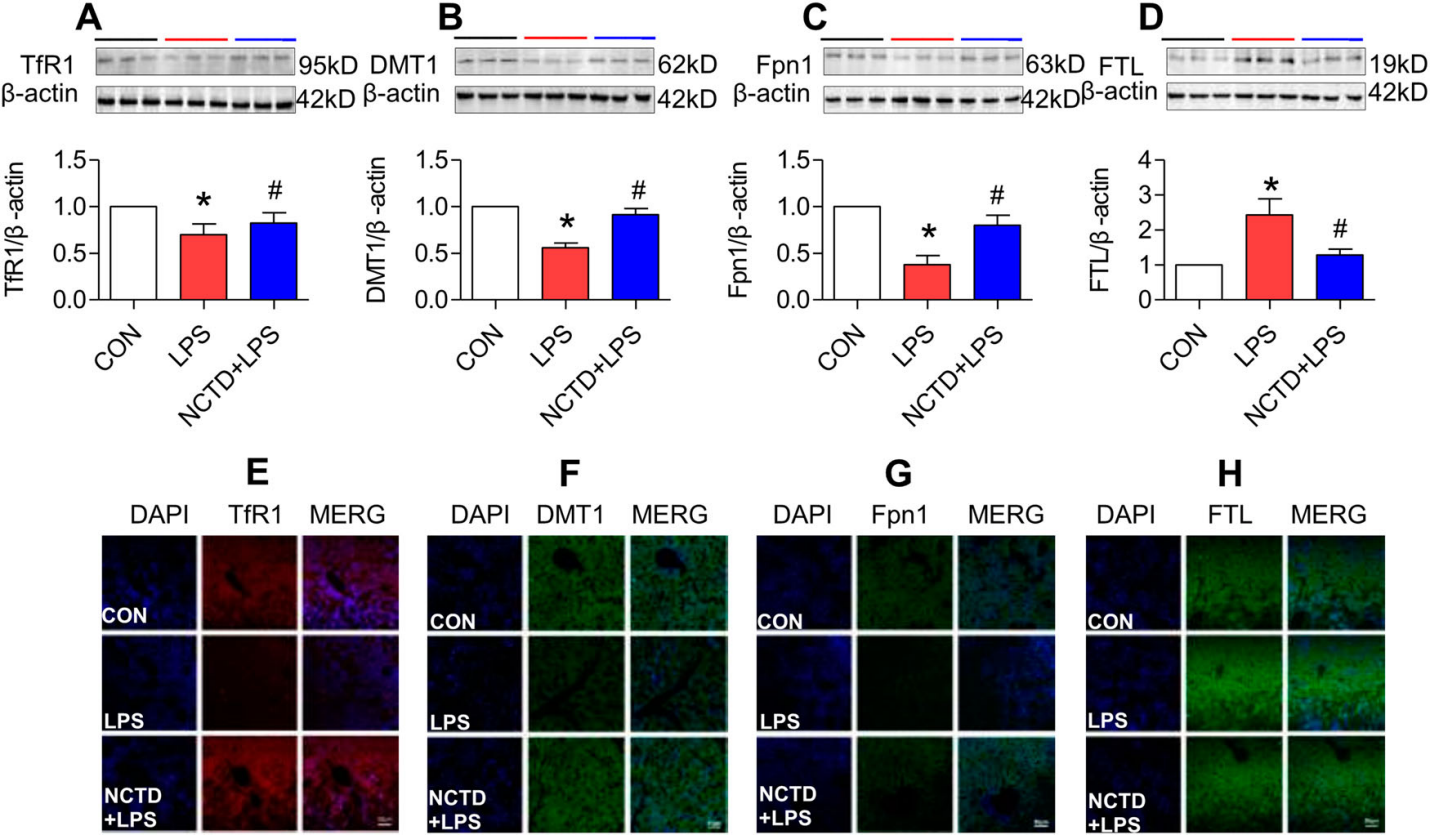
Norcantharidin up-regulated transferrin receptor 1, divalent metal transporter 1, ferroportin 1 expression, and down-regulated ferritin-light chain expression in the liver of lipopolysaccharides-treated mice. The mice in Control, LPS and NCTD + LPS groups were treated as described in ‘Methods’. At 6-h after treatment, the animals were anesthetized and decapitated and the samples collected and expression of TfR1 (A and E), DMT1 (B and F), Fpn1 (C and G) and FTL (D and H) in the liver was measured by Western blot (A-D) and Immunofluorescence staining (E-H) (Scale bar = 50 μm). Data presented as the means ± S.E.M. (*n* = 3) *p < .05 vs. Control. #*p* < .05 vs. LPS.

**Figure 3.**
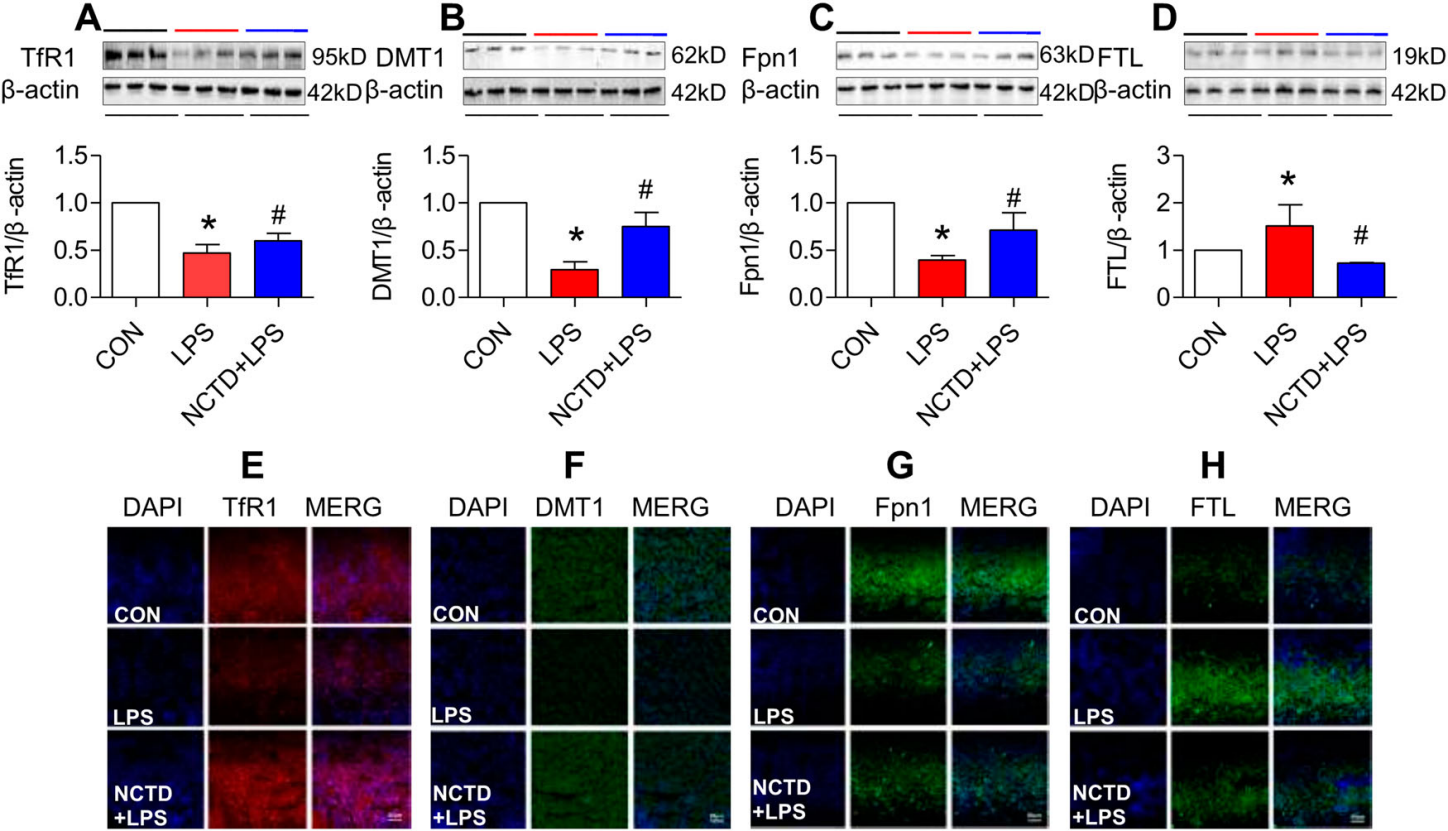
Norcantharidin up-regulated transferrin receptor 1, divalent metal transporter 1, ferroportin 1 expression, and down-regulated ferritin-light chain expression in the spleen of lipopolysaccharides-treated mice. The mice in Control, LPS and NCTD + LPS groups were treated as described in ‘Methods’. At 6-h after treatment, the animals were anesthetized and decapitated and the samples collected and expression of TfR1 (A and E), DMT1 (B and F), Fpn1 (C and G) and FTL (D and H) in the spleen was measured by Western blot (A–D) and Immunofluorescence staining (E–H) (Scale bar = 50 μm). Data presented as the means ± S.E.M. (*n* = 3) *p < .05 vs. Control. #*p* < .05 vs. LPS.

**Figure 4.**
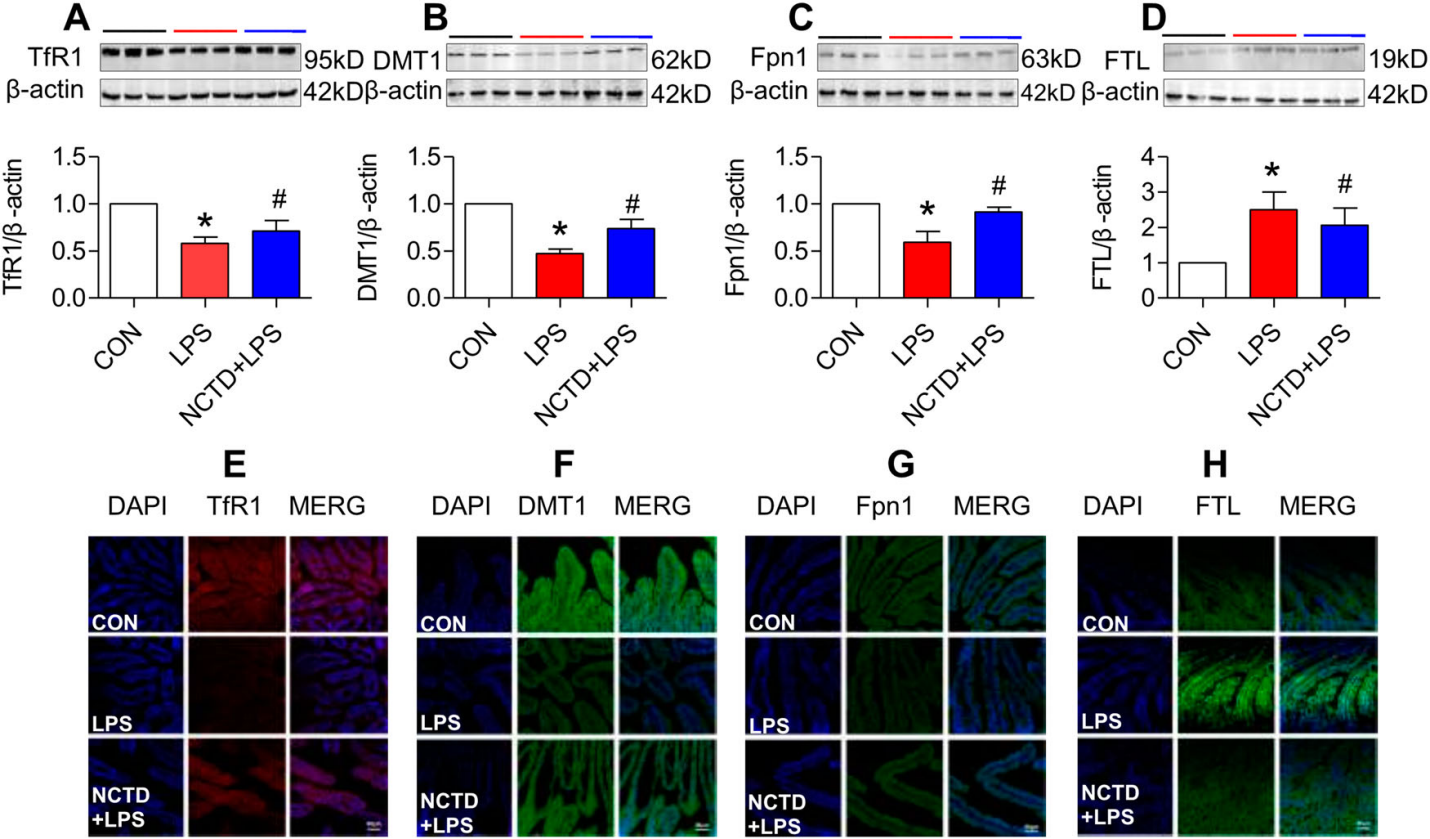
Norcantharidin up-regulated transferrin receptor 1, divalent metal transporter 1, ferroportin 1 expression, and down-regulated ferritin-light chain expression in the duodenum of lipopolysaccharides-treated mice. The mice in Control, LPS and NCTD + LPS groups were treated as described in ‘Methods’. At 6-h after treatment, the animals were anesthetized and decapitated and the samples collected and expression of TfR1 (A and E), DMT1 (B and F), Fpn1 (C and G) and FTL (D and H) in the duodenum was measured by Western blot (A–D) and Immunofluorescence staining (E–H) (Scale bar = 50 μm). Data presented as the means ± S.E.M. (*n* = 3) *p < .05 vs. Control. #*p* < .05 vs. LPS.

To find out the mechanisms by which NCTD affects serum iron, transferrin saturation, and iron content in these organs, we then examined the expression of TfR1, DMT1 and Fpn1, three key proteins in cell-iron uptake [30,31] and release [16,32] in the liver, spleen and duodenum of LPS-treated mice. Western blot analysis showed that treatment with LPS only induced a significant reduction in expression of TfR1 (Figures 2, 3 and 4(A)), DMT1 (Figures 2, 3 and 4(B)) and Fpn1 (Figures 2, 3 and 4(C)) in the liver (Figure 2), spleen (Figure 3) and duodenum (Figure 4) of mice, while the changes induced by LPS were significantly reversed by pre-treatment with NCTD. The expression of (Figures 2, 3 and 4(A)), DMT1 (Figures 2, 3 and 4(B)) and Fpn1 (Figures 2, 3 and 4(C)) in the liver (Figure 2), spleen (Figure 3) and duodenum (Figure 4) was significantly higher in NCTD + LPS-treated mice when compared to LPS-treated mice. Consistent results were also obtained from immunohistochemical examination of TfR1 (Figures 2, 3 and 4(E)), DMT1 (Figures 2, 3 and 4(F)) and Fpn1 (Figures 2, 3 and 4(G)) in the liver (Figure 2), spleen (Figure 3) and duodenum (Figure 4) of mice.

We also examined the effects of NCTD on the expression of FTL in the liver, spleen and duodenum of LPS-treated mice. FTL was checked because FTL facilitates the storage of iron into the ferritin core [33–35], being more closely associated with cellular iron storage [36] and also because FTL is the predominant form of ferritin in the liver and spleen [37,38]. Western blot analysis showed that NCTD could significantly suppress the LPS-induced increase in expression of FTL (Figures 2, 3 and 4(D)) in the liver (Figure 2), spleen (Figure 3) and duodenum (Figure 4) of mice. Also, the results were confirmed by immunofluorescence staining analysis (Figures 2, 3 and 4(H)). These observations provide further evidence that NCTD could down-regulate iron contents in the liver, spleen and duodenum in LPS-treated mice.

### Norcantharidin up-regulated expression of IRP1 in the liver, spleen and duodenum of lipopolysaccharides-treated mice

**Figure 5.**
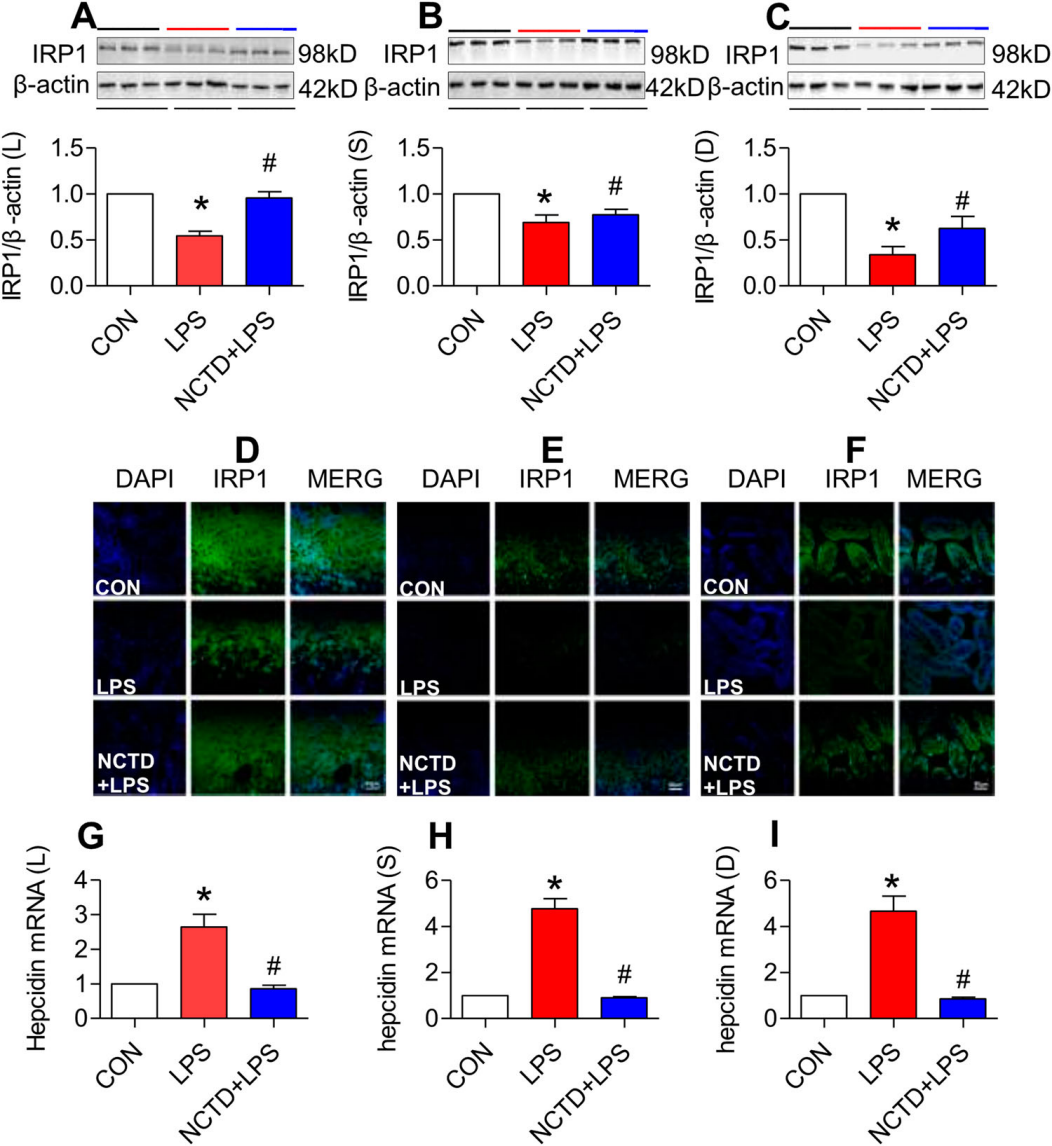
Norcantharidin up-regulated expression of IRP1 and down-regulated hepcidin mRNA in the liver, spleen and duodenum of lipopolysaccharides-treated mice. The mice in Control, LPS and NCTD + LPS groups were treated as described in ‘Methods’. At 6-h after treatment, the animals were anesthetized and decapitated and the samples collected and expression of IRP1 and hepcidin mRNA in the liver (IRP1: A and D, Hepcidin mRNA: G), spleen (IRP1: B and E, Hepcidin mRNA: H) and duodenum (IRP1: C and F, Hepcidin mRNA: I) of lipopolysaccharides-treated mice was measured by Western blot (A–C), Immunofluorescence staining (D–F) (Scale bar = 50 μm) or RT-PCR (G–I). Data presented as the means ± S.E.M. (*n* = 3) *p < .05 vs. Control. #*p* < .05 vs. LPS.

To elucidate how NCTD up-regulates TfR1, DMT1 and Fpn1 expression and down-regulates FTL in LPS-treated mice, we subsequently investigated the effects of NCTD on IRP1 (iron regulatory protein 1) and hepcidin expression in mice because cellular expression of these proteins is regulated by IRPs (iron regulatory proteins) and systemically by hepcidin [39,40]. Western blot (Figure 5(A–C)) and immunofluorescence staining (Figure 5(D–F)) analysis both showed that IRP1 expression in the liver (Figure 5(A,D)), spleen (Figure 5(B,E)) and duodenum (Figure 5(C,F)) was significantly higher in NCTD + LPS-treated mice than in LPS-treated mice, indicating that NCTD could significantly reverse the inhibition of LPS on IRP1 in all organs we examined. RT-PCR analysis demonstrated that expression of hepcidin mRNA in the liver (Figure 5(G)), spleen (Figure 5(H)) and duodenum (Figure 5(I)) was significantly lower in NCTD + LPS-treated mice than in LPS-treated mice, indicating that NCTD has the ability to significantly reverse the promoting role of LPS in the expression of hepcidin mRNA in these organs.

### Norcantharidin down-regulated expression of IL-6, JAK2 and STAT3 in the liver, spleen and duodenum of lipopolysaccharides-treated mice

**Figure 6.**
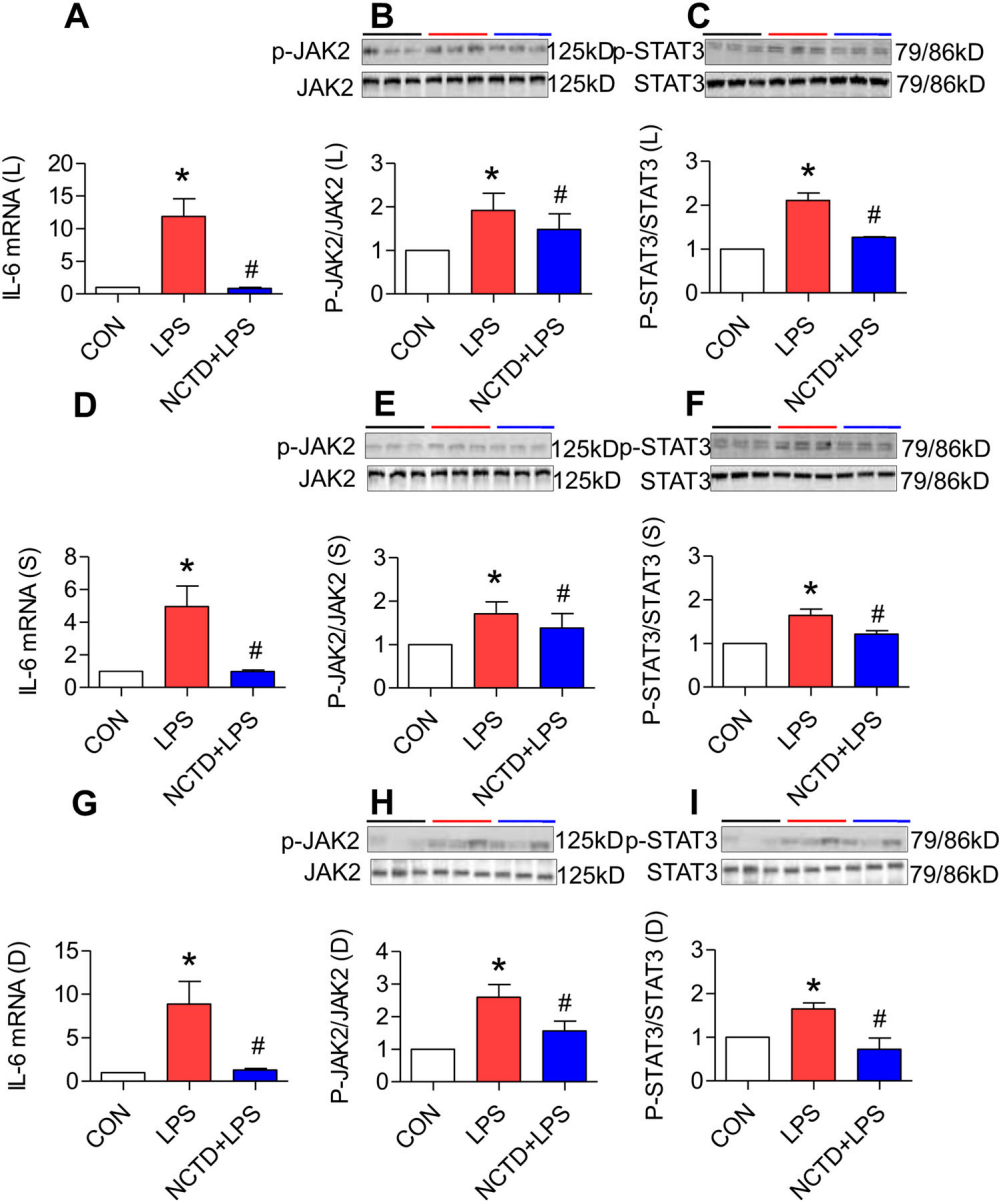
Norcantharidin down-regulated expression of IL-6, JAK2 and STAT3 in the liver, spleen and duodenum of lipopolysaccharides-treated mice. The mice in Control, LPS and NCTD + LPS groups were treated as described in ‘Methods’. At 6-h after treatment, the animals were anesthetized and decapitated and the samples collected and expression of IL-6 mRNA (A, D and G), p-JAK2 (B, E and H) and p-STAT3 (C, F and I) proteins in the liver (A–C), spleen (D–F) and duodenum (G–I) of lipopolysaccharides-treated mice was measured by RT-PCR and Western blot analysis. Data presented as the means ± S.E.M. (*n* = 3) *p < .05 vs. Control. #*p* < .05 vs. LPS.

It has been well-demonstrated that LPS up-regulates hepcidin expression via the IL-6/JAK/STAT3 signaling pathway [13,41]. To explore the possible mechanisms involved in the effect of NCTD on hepcidin, we next investigated the roles of NCTD in the expression of IL-6 mRNA, p-JAK2 and p-STAT3 proteins in LPS-treated mice. It was found that treatment with LPS only induced a significant increase in the expression of IL-6 mRNA (Figure 6(A,D,G)), p-JAK2 (Figure 6(B,E,H)) and p-STAT3 (Figure 6(C,E,I)) proteins in the liver (Figure 6(A–C)), spleen (Figure 6(D–F)) and duodenum (Figure 6(G–I)) of mice. Pre-treatment with NCTD led to a significant reduction in the expression of IL-6 mRNA (Figure 6(A,D,G)), p-JAK2 (Figure 6(B,E,H)) and p-STAT3 (Figure 6(C,E,I)) proteins in the liver (Figure 6(A–C)), spleen (Figure 6(D–F)) and duodenum (Figure 6(G–I)) of LPS-treated mice, showing a significant reversing role of NCTD on the impact of LPS on the expression of IL-6, p-JAK2 and p-STAT3 in these organs.

## Discussion

In the present study, we demonstrated that NCTD could significantly up-regulate serum iron level and Tf saturation (%), expression of TfR1, DMT1, Fpn1 and IRP1 proteins, and down-regulate iron contents, expression of FTL, pJAK2 and pSTAT3 proteins and hepcidin and IL-6 mRNAs in the liver, spleen and duodenum of LPS-treated mice. These findings indicate that NCTD is capable of suppressing the effects of LPS or infection and inflammation on iron metabolism, and also provide in vivo evidence for the usefulness of NCTD for iron homeostasis under inflammatory conditions.

**Figure 7.**
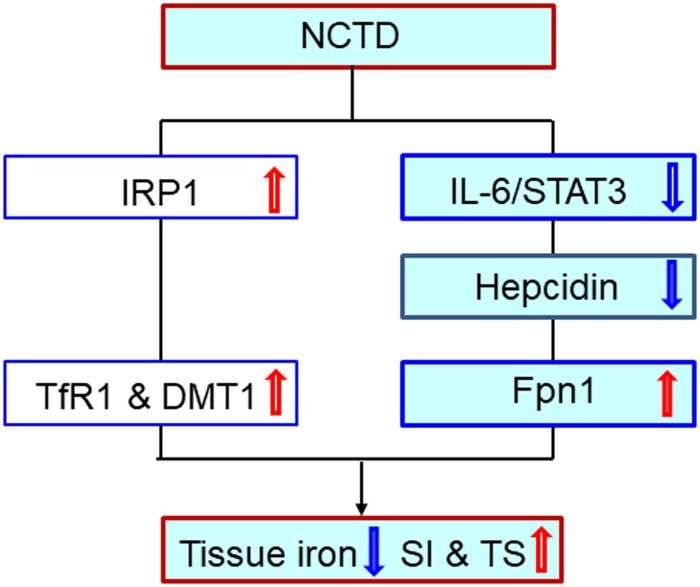
A hypothetical scheme for the role of Norcantharidin in the regulation of serum iron and transferrin saturation and iron content in liver, spleen and duodenum of LPS-treated mice.

The significant reduction in iron contents induced by NCTD in the liver, spleen and duodenum of LPS-treated mice may be due to the NCTD-induced up-regulation of Fpn1 expression because increased Fpn1 would increase the amount of iron exported from the cells. In most types of cells throughout the body, iron balance depends on the normal expression of in-and-out transporters of iron, TfR1, DMT1 and Fpn1 [16,30–32,42]. By controlling their expression, the cell can determine the amount of iron acquired (via TfR1 and DMT1) and released (via Fpn1). In the present study, however, in addition to the increased Fpn1, we also found that NCTD induces a significant increase in the expression of TfR1 and DMT1 in the organs of LPS-treated mice. The increased expression of these two iron importers should lead to increased iron import and contents in the cells or tissues. However, iron contents were found to be reduced in the organs we examined. This may indicate that the effect of NCTD on Fpn1 expression is more significant than its effect on TfR1 and DMT1. In other words, the increased amount of iron released from the cells via Fpn1 is more than that of iron imported into the cells via TfR1 and DMT1 under our experimental conditions (Figure 7).

It has been well-confirmed that TfR1, DMT1 and Fpn1 expression is mainly controlled by IRPs [43–45] as well as hepcidin [39,40,46,47]. The expression of TfR1, DMT1 and Fpn1 is coordinately regulated by the IRP/IRE (iron-responsive element) system, [39,40,48,49] positively for the 3′ untranslated region IRE motif in TfR1 and DMT1 and negatively for the 5′ untranslated region IRE motif in Fpn1 (and FTL), namely to up-regulate TfR1 and DMT1 and down-regulate Fpn1 (and FTL) expression. Hepcidin reduces the *amount of* Fpn1 on the membrane by directly binding with Fpn1, and the hepcidin/Fpn1 complex is then internalized and subsequently degraded [50]. It has also been reported that hepcidin is capable of directly inhibiting TfR1 in different types of cells [46–48] and down-regulating DMT1 expression in the intestine through proteasome internalization and degradation [51]. Therefore, the increased TfR1 and DMT1 caused by NCTD may be due to NCTD-induced up-regulation in IRP1 and down-regulation in hepcidin, while the increased Fpn1 is likely a result mainly of the down-regulation in hepcidin in the liver, spleen and duodenum of mice co-treated by LPS + NCTD.

Hepcidin is the master regulator in systemic iron metabolism, and hepcidin expression is regulated by a variety of factors, such as systemic iron levels [52] and inflammation [53]. Inflammation has been well-documented to affect iron metabolism via hepcidin. It has been demonstrated that LPS is able to up-regulate hepcidin expression via the IL-6/JAK2/STAT3 signaling pathway [17]. In the mice co-treated with NCTD and LPS, the expression of IL-6 mRNA, p-JAK2 and p-STAT3 proteins and also hepcidin mRNA was found to be significantly reduced in the liver, spleen and duodenum, as compared with mice treated with LPS only. This clearly indicated that NCTD down-regulates the expression of hepcidin by inhibiting the IL-6/JAK2/STAT3 signaling pathway in mice under inflammatory conditions.

It has been proved that NCTD, as an anti-tumor drug, can inhibit the growth and metastasis of tumor cells in vivo and in vitro [54,55]. Due to rapid proliferation and DNA synthesis, tumor cells demand more iron than normal cells. Iron plays an important role in matrix degradation and tumor metastasis by stimulating or stabilizing the activity of certain metalloproteinases [56]. Increased iron levels in the body have been considered as an important cofactor in the carcinogenesis of several cancers [57]. This notion was supported by most studies in which increased levels of iron have been shown to be associated with higher cancer risk [58–60]. In the present study, NCTD was found to have the ability to reduce tissue iron contents in LPS-treated mice. This may be a novel mechanism associated with the anti-cancer effects of NCTD. Further studies about this possibility are needed.

Anemia of inflammation (AI, or ACD anemia of chronic disease) is considered the second most prevalent type of anemia worldwide, after iron deficiency anemia (IDA) and the most frequent anemic entity observed in hospitalized or chronically ill patients [18,61,62]. In animal models of AI and in patients suffering from inflammatory diseases, increased hepcidin levels are associated with low Fpn1 expression on duodenal enterocytes and macrophages, along with impaired dietary iron absorption and retention of iron in macrophages [63,64], thereby causing decreased serum iron, transferrin saturation, and iron delivery for erythropoiesis. Therefore, hepcidin measurement has been considered as a promising diagnostic tool for AI or ACD [64–66]. In the present study, we showed that NCTD could significantly suppress the LPS-induced increase in hepcidin expression and decrease in serum iron and transferrin saturation, indicating that NCTD may have a potential role in the treatment of AI as an ancillary drug.

In summary, we demonstrated that NCTD could increase serum iron and Tf saturation and reduce tissue iron and FTL contents by affecting the expression of cell-iron transport proteins TfR1, DMT1 and Fpn1. Our findings also revealed that the impact of NCTD on the expression of TfR1, DMT1 and Fpn1 is mediated by up-regulating IRP1 and down-regulating hepcidin expression, while down-regulation of NCTD on the expression of hepcidin is mediated by the IL-6/JAK2/STAT3 signaling pathway in LPS-treated mice (Figure 7). Our findings provide further evidence in vivo to support the conclusion that NCTD modifies iron metabolism by affecting the expression of IL-6/JAK2/STAT3 /hepcidin and IRP1. The ability of NCTD to suppress the LPS-induced increase in hepcidin expression and decrease in serum iron and transferrin saturation implies that NCTD may have a potential role in the treatment of AI as an ancillary drug. The findings also suggest that reducing tissue iron contents may be a novel mechanism associated with the anti-cancer effects of NCTD. Further studies about this possibility are needed.

## Data Availability

The datasets used and/or analyzed during the current study are available from the corresponding author on reasonable request.

## Abbreviations

Abbreviations: ACD: anemia of chronic disease; AI: anemia of inflammation; BCa: breast cancer; CIA: collagen-induced arthritis; CTD: cantharidin; DMT1: divalent metal transporter1; EMT: epithelial–mesenchymal transition; Fpn1: ferroportin 1; FTL: ferritin-light chain; IL-6: interleukin-6; IRP1: iron regulatory protein 1; JAK2: Janus kinase 2; LPS: lipopolysaccharide; NCTD: Norcantharidin; PBS: phosphate-buffered saline; SI: serum iron; STAT3: signal transducer and activator of transcription 3; TF% saturation: transferrin saturation; TfR1: transferrin receptor 1; UIBC: unsaturated iron-binding capacity; UUO: unilateral ureteral obstruction
